# A practical guide to male hypogonadism in the primary care setting

**DOI:** 10.1111/j.1742-1241.2010.02355.x

**Published:** 2010-05

**Authors:** P Dandona, M T Rosenberg

**Affiliations:** 1 Division of Endocrinology, Diabetes and Metabolism, State University of New York at Buffalo and Kaleida HealthBuffalo, NY, USA; 2 Mid Michigan Health CentersJackson, MI, USA

## Abstract

There is a high prevalence of hypogonadism in the older adult male population and the proportion of older men in the population is projected to rise in the future. As hypogonadism increases with age and is significantly associated with various comorbidities such as obesity, type 2 diabetes, hypertension, osteoporosis and metabolic syndrome, the physician is increasingly likely to have to treat hypogonadism in the clinic. The main symptoms of hypogonadism are reduced libido/erectile dysfunction, reduced muscle mass and strength, increased adiposity, osteoporosis/low bone mass, depressed mood and fatigue. Diagnosis of the condition requires the presence of low serum testosterone levels and the presence of hypogonadal symptoms. There are a number of formulations available for testosterone therapy including intramuscular injections, transdermal patches, transdermal gels, buccal patches and subcutaneous pellets. These are efficacious in establishing eugonadal testosterone levels in the blood and relieving symptoms. Restoration of testosterone levels to the normal range improves libido, sexual function, and mood; reduces fat body mass; increases lean body mass; and improves bone mineral density. Testosterone treatment is contraindicated in subjects with prostate cancer or benign prostate hyperplasia and risks of treatment are perceived to be high by many physicians. These risks, however, are often exaggerated and should not outweigh the benefits of testosterone treatment.

**Review Criteria**

- We summarise information that primary care physicians need to know about this common condition, found in 20–40% of older men.
- Articles were identified from MEDLINE in May–September 2009 (search limits; last 10 years, humans, English language) using the terms testosterone and hypogonadism.
- In general, we cite articles that review a broad range of hypogonadal comorbidities as well as professional societies’ guidelines for testosterone diagnosis, treatment options and monitoring procedures.

**Message for the Clinic**

- Physicians must be alert to the fact that many of their patients may be suffering from low testosterone levels.
- Hypogonadism increases with age and is particularly associated with some of the most common conditions found in patients visiting primary care clinics, such as diabetes and obesity.
- Physicians need to know the symptoms of hypogonadism and the various testosterone replacement treatment options.

## Introduction

There is a high prevalence of hypogonadism in the middle- and older-aged male population and various prevalence figures have been described in a number of studies. A consistent feature of these studies is that hypogonadism increases with age. As the number of individuals aged 65 years and over in the US population is projected to rise from approximately 40 million (13.0%) in 2010 to approximately 60 million (17.9%) in 2025, the number of men, who presently comprise approximately 43% of this population, that are hypogonadal will also increase (1). As a result, physicians will be increasingly likely to encounter men with the symptoms of hypogonadism in the clinic.

The purpose of this review is to summarise the current understanding of male hypogonadism, with particular reference to the needs of the primary care physician. A key consideration for any physician is to understand the clinical significance of low testosterone levels and how hypogonadal men are likely to benefit from testosterone replacement therapy. It is also important to have a good understanding of the contraindications and risks associated with the therapy as well as the necessary treatment monitoring required.

## Pathophysiology

The Endocrine Society defines male hypogonadism as a clinical syndrome that results from failure of the testis to produce physiological levels of testosterone (androgen deficiency) and the normal number of spermatozoa caused by disruption of one or more levels of the hypothalamic–pituitary–gonadal (HPG) axis (2).

There are other definitions published by the American Association of Clinical Endocrinologists (AACE) (3) and the various international and European societies of andrology and urology (4). The condition has a number of different names reflecting differing opinions (5) (Table 1). The different names have arisen as authors try to separate the hypogonadism resulting from natural ageing from, for example, the hypogonadism caused by testicular trauma. In this review, hypogonadism will be used as a general term to refer to any state characterised by low blood testosterone levels.

**Table 1:** Alternative names for male hypogonadism (2,4,77,98)

Androgen deficiency syndrome
Androgen deficiency in the ageing male (ADAM)
Andropause
Late-onset hypogonadism
Male menopause
Partial androgen decline in the ageing male (PADAM)
Testosterone deficiency syndrome

### Testosterone regulation in the eugonadal male

**Figure 1:**
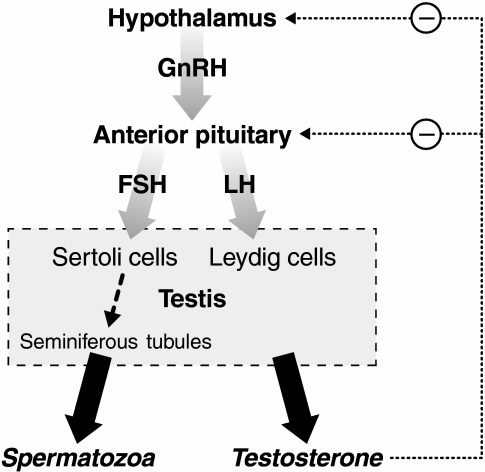
The hypothalamic–pituitary–gonadal axis in men. GnRH, FSH, and LH have stimulatory effects on their targets. Testosterone inhibits their release from the hypothalamus and the pituitary. FSH, follicle-stimulating hormone; GnRH, gonadotropin-releasing hormone; LH, luteinizing hormone

Androgens are vital for the maintenance and development of sexual function in men. The regulation of testosterone production in eugonadal men depends on the HPG axis depicted in Figure 1. The hypothalamus secretes gonadotropin-releasing hormone (GnRH) that acts on the anterior pituitary to produce follicle-stimulating hormone (FSH) and luteinizing hormone (LH). LH acts on the interstitial Leydig cells of the testes, stimulating them to produce testosterone, whereas FSH stimulates spermatogenesis and Sertoli cell function (6,7).

Secretion of LH from the pituitary is not constant, but has approximately six bursts of secretion per day with an early morning high and an early evening low. A total of approximately 7 mg of testosterone is secreted each day (8), although in older men the rate decreases (9).

Only 1–2% of testosterone circulates free in the blood; the remaining 98–99% is bound to albumin (40–50%) and to sex hormone binding globulin (SHBG) (50–60%). Testosterone binds strongly to SHBG, and it is therefore largely the free and albumin-bound testosterone that is available for biological action (10). For this reason, free and albumin-bound testosterones together are termed bioavailable testosterone (BAT).

The HPG axis is regulated by a negative feedback mechanism (Figure 1). Testosterone inhibits the frequency and amplitude of GnRH release from the hypothalamus and also the secretion of LH from the pituitary. The Sertoli cells of the testes, in addition to stimulating spermatogenesis, also secrete the glycoprotein hormone inhibin, which provides negative feedback to the pituitary, inhibiting the secretion of FSH (11).

Testosterone is converted to dihydrotestosterone (DHT) by 5α-reductase enzymes or to estradiol by aromatase in target cells. Testosterone and DHT both bind to the androgen receptor where they exert their biological effects. Approximately 20% of the DHT in the circulation is produced directly by testicular secretion, with the remaining 80% being derived from conversion from testosterone in the peripheral tissues (10).

Target cells that contain 5α-reductase are concentrated in the prostate, reproductive system and skin. Aromatase-containing cells predominate in the liver and adipose and regions of the brain (6,12).

Testosterone and other androgens have important biological and physiological effects, summarised in Table 2. Apart from the vital role that it plays during puberty in stimulating the development of male secondary sexual characteristics and their maintenance thereafter, it has multiple other physiological effects. These include the promotion of haemoglobin synthesis and red blood cell production; the stimulation of anabolic muscular development and bone growth; and the suppression of adipose tissue formation. Testosterone also stimulates the basal metabolic rate (8) and has positive effects on mood and cognitive ability (13). Behavioural effects include mediating sexual behaviour and competitive encounters (14), for example, a connection between financial profits and raised endogenous testosterone levels has been described for male commodity traders in the City of London (15).

**Table 2:** Physiological effects of testosterone in male adults (6)

Maintains reproductive tissues
Stimulates spermatogenesis
Stimulates and maintains sexual function
Increases body weight and nitrogen retention
Increases lean body mass
Maintains bone mass
Promotes sebum production, and axillary and body hair growth
Stimulates erythropoiesis

### Primary hypogonadism

Primary hypogonadism is caused by testicular failure and is characterised by low serum testosterone and high LH and FSH concentrations. For this reason, primary hypogonadism is also known as *hypergonadotropic hypogonadism*. Primary hypogonadism can result from testicular injury, tumour, or infection; genetic defects affecting testicular development (e.g. Klinefelter syndrome), as well as chemotherapy, radiation treatment or alcohol abuse (3,16).

### Secondary hypogonadism

In secondary hypogonadism (*hypogonadotropic hypogonadism*), defects in the hypothalamus or pituitary result in low testosterone levels because of insufficient stimulation of the Leydig cells. It is also associated with low or low-normal FSH and LH levels. Patients with secondary hypogonadism can have their fertility restored by suitable hormonal stimulation, whereas those with primary hypogonadism resulting from testicular failure cannot. Secondary hypogonadism can be caused by a number of conditions (Table 3) including hypothalamic and pituitary disorders or lesions, hyperprolactinemia and Kallmann syndrome (which causes a GnRH deficiency) (16). Certain medications and illnesses can also affect the hypothalamic–pituitary system resulting in hypogonadism (17).

**Table 3:** Causes of male hypogonadism (3,16,76)

**Primary hypogonadism**
Congenital anorchidism
Cryptorchidism
Mumps orchitis
Genetic and developmental conditions: Klinefelter syndrome,androgen receptor and enzyme
Defects, Sertoli cell only syndrome
Radiation treatment/chemotherapy
Testicular trauma
Autoimmune syndromes (anti-Leydig cell disorders)
**Secondary hypogonadism**
Genetic conditions: Kallmann’s syndrome, Prader-Willisyndrome
Pituitary tumours, granulomas, abscesses
Hyperprolactinemia
Cranial trauma
Radiation treatment
Various medications
Mixed (primary and secondary) hypogonadism*
Alcohol abuse
Ageing
Chronic infections (HIV)
Corticosteroid treatment
Hemochromatosis
Systemic disease (liver failure, uremia, sickle-cell disease)

* Mixed hypogonadism is often included within the secondary hypogonadism category.

It should be noted that low testosterone can be caused by a combination of both primary and secondary hypogonadism (also called *mixed hypogonadism*) that reflects defects in the hypothalamus and/or the pituitary as well as the testes. This is found in men with sickle-cell disease, thalassemia, alcoholism, glucocorticoid treatment, and in older men (2). In general, however, the term mixed hypogonadism is not used in clinical practice in the US and is considered part of secondary hypogonadism.

## Prevalence and comorbidities

### Prevalence of hypogonadism

In the Baltimore Longitudinal Study on Ageing, it was found that 19% of men over 60 years had low testosterone. The Hypogonadism in Males (HIM) study estimated the overall prevalence of hypogonadism at approximately 39% in men aged 45 years or older (18). It has been estimated that only 5–35% of hypogonadal males actually receive treatment for their condition (19,20).

Measuring testosterone levels in populations, while useful, is different from measuring hypogonadal symptoms. A subject can have low testosterone levels, but can also have no clinically significant symptomatology. Likewise, measurement of symptoms alone is not reliable, as hypogonadal symptoms are non-specific. The Massachusetts Male Ageing Study (MMAS) measured a combination of testosterone levels and hypogonadal symptoms and found between 6% and 12% of men had symptomatic androgen deficiency (21). An interesting observation from the MMAS was that half of the men found to have symptomatic androgen deficiency at one stage were found to be eugonadal when retested at a later stage (22). This is probably because there is subject-to-subject variation in testosterone secretion and in the testosterone threshold where symptoms become manifest. As discussed below, a measurement of low testosterone in a patient should be reconfirmed at a later stage before considering treatment.

Older men are more likely to have low testosterone levels: in the HIM study, for example, the prevalence of low testosterone in the 45–54 age group was 34%, whereas it was 50% in men over 85 years (18). Likewise, in the Baltimore study the percentage of men with low testosterone increased from 12% in men in their 50s, to 49% in men over 80 years of age (23).

There appears to be no consistent evidence that the prevalence of hypogonadism differs between racial and ethnic groups (24–26).

### Common comorbidities

Higher rates of hypogonadism than those in the general population are associated with various common diseases or conditions. The HIM study calculated odds ratios for some common conditions (Table 4). It is not yet understood whether the low testosterone levels are a consequence of the disease, are connected with the disease’s aetiology, or are one of the causes of the disease. Further studies are needed to determine if treating the associated hypogonadism is likely to improve the patient’s disease symptoms.

**Table 4:** Odds ratios for hypogonadism for various comorbidities from the HIM Study (18)

Condition	Odds ratio
Obesity	2.38
Diabetes	2.09
Hypertension	1.84
Hyperlipidaemia	1.47
Osteoporosis	1.41
Asthma/chronic obstructive pulmonary disease	1.40

#### Cardiovascular disease

Vascular tissue (including endothelium and vascular smooth muscle cells) contains androgen receptors, so it is to be expected that testosterone (or its metabolite, oestrogen) is likely to affect the cardiovascular system. In fact, the increased risk of cardiovascular disease in males compared with females has been taken to imply a role for testosterone (or oestrogen) in the disease. Human observational studies, however, have shown no associations between high testosterone levels and coronary artery disease, and testosterone has been shown to dilate the coronary arteries both *in vitro* and *in vivo*. Some studies have shown that testosterone can be considered to have a positive effect on reducing the risk factors for cardiovascular disease; for example, inverse relationships have been shown between testosterone levels and body mass index (BMI), waist circumference, waist-hip ratio, serum leptin, low-density lipoprotein (LDL) cholesterol, triglyceride and fibrinogen levels. Low testosterone is associated with dyslipidemia, hypertension, obesity and diabetes, all of which increase the risk of cardiovascular disease and are features of the metabolic syndrome (27,28).

A negative view of testosterone’s impact on cardiovascular disease comes from the observation that high-density lipoprotein (HDL) cholesterol levels decrease in patients on oral testosterone therapy, or when taken in supraphysiological doses by athletes (29,30). However, when given as a transdermal gel to hypogonadal men, there is either no significant change or only minor changes in HDL levels (28,31,32). Whatever the subtleties of the effects of testosterone on lipids, recent data have demonstrated that low testosterone concentrations are associated with an increased incidence of cardiovascular events, and an increase in acute myocardial infarction and stroke.

The relationship between testosterone and HDL is confounded by the fact that both HDL and testosterone are inversely related to BMI. In fact, epidemiological analyses have found that HDL levels are positively linked to testosterone levels in middle-aged men. Data from the MMAS have demonstrated that there is a strong, positive relationship between HDL and testosterone in men with cardiovascular disease (low total or free testosterone correlates with low HDL cholesterol) (31).

Recent work in the Rancho Bernardo, California population has shown that men with serum total testosterone levels in the lowest quartile (< 241 ng/dl) were associated with a higher risk (38%) of cardiovascular mortality, compared with those who have higher total testosterone levels, independent of age, obesity and lifestyle choices (33). In fact, those with low testosterone were 40% more likely to die (all-cause mortality) than those with higher levels. This is in contrast to what was found in the MMAS study where total testosterone levels were unrelated to all-cause mortality (34,35).

A meta analysis, published in 2007, of randomised trials that assessed the effect of exogenous testosterone on cardiovascular events, however, concluded that the inference that testosterone use in men is not associated with important cardiovascular effects was only weakly supported. Large randomised trials using men with and without cardiovascular disease and with cardiovascular end-points are needed to better assess the consequences of testosterone treatment on cardiovascular risk (36).

A recent study (2009) from Italy demonstrates that testosterone treatment in elderly patients with chronic heart failure improves insulin sensitivity and various cardiorespiratory and muscular outcomes (37).

#### Diabetes

In 2007, it was estimated that 23.6 million people, or 7.8% of the US population, had diabetes (38). Projections based on data from the National Health and Nutrition Examination Surveys (NHANES) show that by 2021 there are anticipated to be approximately 33 million people with diabetes in the United States, representing 13.5% of the population (39).

Low testosterone concentrations are known to occur in association with type 2 diabetes. However, clinicians have often not related low testosterone concentrations to clinical hypogonadism. The first attempt to measure free testosterone and to establish hypogonadism as a feature of male type 2 diabetes was made by Dhindsa et al. in 2004 (40). This has been confirmed in several other studies including the HIM study (41). In the HIM study, a diabetic man was approximately twice as likely to be hypogonadal compared with a non-diabetic man (18). Prevalence in diabetic men has been estimated at 33–50% (18,40,42). With such a high prevalence, hypogonadism is a candidate for the most common complication of male type 2 diabetes. Analysis of gonadotropin levels demonstrates that the hypogonadism in type 2 diabetes is mostly hypogonadotropic (secondary) hypogonadism (40). There is no relation between the degree of hyperglycaemia and testosterone concentration (40,43).

C-reactive protein, a marker for systemic inflammation, has been found to be markedly elevated in patients with secondary hypogonadism and type 2 diabetes. The concentrations of C-reactive protein in these patients are twice as high as those in eugonadal type 2 diabetics, whose C-reactive protein levels are already elevated compared with non-diabetics. Such patients have also been shown to have mild anaemia, low bone mineral density (BMD) in the arms and ribs, and increased adiposity when compared with eugonadal type 2 diabetics (44,45). These features are similar to those of hypogonadal patients without diabetes. Another intriguing observation is that prostate-specific antigen (PSA), a marker for prostate cancer, is significantly lower in type 2 diabetics and this is related to their lower plasma testosterone concentrations (46). The clinical significance of this remains to be elucidated.

Interestingly, low testosterone concentrations predict the development of type 2 diabetes. Utilising data from the NHANES III survey, it was found that men in the lowest free testosterone tertile were four times as likely to have diabetes as those in the highest free testosterone tertile (47).

Type 1 diabetes does not seem to be associated with hypogonadism, suggesting that hypogonadism is specific to type 2 diabetes and not related specifically to hyperglycaemia (43).

#### Obesity and the metabolic syndrome

Obesity amongst adult men had a prevalence of 33.3% in 2005–2006 according to the most recent NHANES review (48). An adult who has a BMI between 25 and 29.9 kg/m^2^ is considered overweight, whereas an adult who has a BMI of 30 kg/m^2^ or higher is considered obese. This is not a rigid rule as BMI does not directly measure body fat, so athletes, for example, may have high BMIs even though they are not overweight (49).

The health risks associated with obesity are well known, increasing the risk for type 2 diabetes, hypertension, atherosclerotic diseases and coronary heart disease. Obesity is strongly associated with type 2 diabetes: approximately 83% of diabetic patients are overweight or obese (50). Obesity is also associated with low total testosterone and reduced SHBG levels. There is an inverse linear relationship between total testosterone and BMI, and free testosterone concentrations also decrease with increasing BMI. There is an inverse relationship between serum total and free testosterone levels and visceral fat mass. Thus, the degree of hypogonadism is positively correlated to the degree of obesity in obese men (51,52).

A person with metabolic syndrome is defined as having central obesity in addition to any two of these four factors: hypertension (≥ 130/85 mm Hg), reduced HDL (< 40 mg/dl in males), raised triglycerides (≥ 150 mg/dl) or raised fasting plasma glucose (≥ 100 mg/dl)(53). It is considered a high risk for coronary heart disease (19,54). As the constituent elements of metabolic syndrome are themselves correlated with testosterone concentrations, it is perhaps not surprising that hypogonadism is also associated with the metabolic syndrome, as has been shown in a number of epidemiological studies (55,56).

Low testosterone levels are correlated with insulin resistance in both epidemiological and interventional studies, and this may be attributable to the effect of testosterone on adiposity. Low testosterone levels increase fat mass and decrease lean muscle, resulting in increased adipose tissue (52).

**Figure 2:**
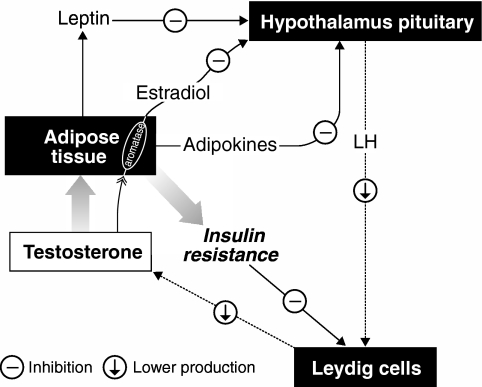
The interrelationship between hypogonadism and insulin resistance (after (42,51)). LH, luteinizing hormone. Low testosterone stimulates an increase in adiposity. Adipose tissue contains high concentrations of aromatase, which reduces testosterone concentrations by converting it to estradiol. The estradiol negatively feeds back on the HPG system, reducing testosterone production in the Leydig cells. Increasing adipose tissue increases insulin resistance, which negatively impacts the Leydig cells as well as inhibiting the release of luteinizing hormone (LH) via the release of adipokines (inflammatory cytokines) such as TNF-α. Leptin, released in response to increased adiposity, also inhibits the release of LH via its effect on the release of gonadotropin-releasing hormone

Adipose tissue affects testosterone levels by increasing the aromatisation of testosterone to estradiol, because the aromatase enzyme is concentrated in adipocytes. This reduces serum and tissue testosterone levels. The estradiol produced by aromatisation also provides negative feedback on the HPG axis, further reducing testosterone. Thus, adiposity potentially leads to hypogonadism, which itself promotes further adiposity. Figure 2 illustrates the main hypogonadal-obesity-insulin resistance connections and also includes other factors such as TNF-α (an adipokine), which is elevated in obese males (42,51,57,58).

However, it seems likely that testosterone may suppress insulin resistance independently of its effects on adiposity. The withdrawal of testosterone therapy in hypogonadal patients that had been stabilised on this therapy leads to an increase in insulin resistance within 2 weeks and prior to significant weight gain (59). A recent study showed that supervised diet and exercise increased testosterone levels in hypogonadal men with metabolic syndrome and newly diagnosed type 2 diabetes. In addition, a small dose (50 mg/day) of testosterone gel improved both glycemic control and insulin sensitivity over and above the improvements because of diet and exercise (60). The mechanism underlying the insulin sensitising effects of testosterone needs to be elucidated.

An important proviso to this discussion is that further research into the role of hypogonadism in obesity, metabolic syndrome and diabetes is required to gain a better understanding of the pathogenic mechanisms involved and that, at present, it is not known whether hypogonadism is the cause or the consequence of these conditions.

#### Osteoporosis

Osteoporosis is an under-recognised problem in men. Ten to twenty per cent of individuals with osteoporosis over 50 years old in the United States are men (25). Up to 13 million men are at increased risk because of low BMD and up to 2 million of these have osteoporosis (61,62). Men have almost 30% of all hip fractures and men are twice as likely to die in hospital than women after a hip fracture. Hip fracture incidence is low until after 75 years, when the risk increases exponentially. Vertebral fractures are also common, although they are only about half as common in men compared with women (63).

There are many suspected causes of osteoporosis, and the most frequent are corticosteroid use, Cushing’s syndrome, hypogonadism and excessive alcohol consumption. In a study of elderly men in a nursing home who have experienced hip fractures, 66% were hypogonadal (64).

Other common secondary causes are smoking, low calcium intake and vitamin D deficiency or insufficiency (61).

Various epidemiological studies in men have examined associations between testosterone and estradiol levels and BMD. Estradiol levels in men have been consistently and positively associated with BMD. Testosterone is also positively associated with BMD, but the relationship is weaker than that of estradiol (65,66).

Interventional studies have shown that testosterone replacement therapy in hypogonadal males increased spine BMD and trabecular connectivity (61,67). However, studies of testosterone therapy in men with osteoporosis are limited and none have used fractures as an end-point; so although there is significant evidence of an association between hypogonadism and osteoporosis, there is no established causal link between the two.

### Complications of treatment for other conditions

#### Corticosteroid treatment

Testosterone levels are lower in men being treated with corticosteroids. Systemic glucocorticoids can reduce testosterone biosynthesis in the testis; in addition, glucocorticoids impact the HPG axis by inhibiting the release of LH (17,68). As a result, patients being treated with glucocorticoids for such chronic conditions as rheumatoid and osteoarthritic inflammation, skin inflammations, asthma, chronic obstructive pulmonary disease (COPD) and inflammatory bowel disease are at an increased risk of hypogonadism.

There have been some studies that suggest that COPD patients have a higher incidence of hypogonadism than the general population and that glucocorticoid treatment is only part of the reason. The pathophysiology of this remains unclear, but suggestions have been made that it might be connected with chronic hypoxia, and a systemic inflammatory response (68). A number of studies have shown that testosterone therapy can improve lean body mass and BMD and strength in hypogonadal men with COPD (17).

#### Chronic pain treatment

Long-acting opioids such as methadone, morphine sulphate, fentanyl and oxycodone for the treatment of chronic pain often result in opioid-induced androgen deficiency (OPIAD). In a case–control study of 40 cancer survivors it was found that 90% of those on opioid treatment were hypogonadal compared with only 40% of the control group (69). The mechanism for OPIAD is thought to involve suppression of GnRH release by the hypothalamus, thereby inducing secondary hypogonadism (17,70).

#### Cancer

Apart from the effects of testicular cancers, which may have a direct impact on testosterone secretion, the prolonged radiation treatment, chemotherapy using antimitotic drugs or corticosteroids or pain treatment medications characteristic of cancer treatment are likely to induce hypogonadism (17). These drugs may induce Leydig cell dysfunction or germinal epithelial failure (71). The HPG axis may also be affected by androgen-, or ectopic adrenocorticotropin hormone-producing tumours, leading to secondary hypogonadism (17).

#### HIV

Androgen deficiency is strongly associated with AIDS wasting syndrome, and testosterone therapy in HIV-positive hypogonadal men increases lean body and muscle mass and perceived well-being, and decreases depression (72–74). Approximately 20–50% of HIV-infected men receiving highly active antiretroviral therapy are hypogonadal. While these prevalence levels may superficially appear similar to the background figures in the population, most studies are based on middle-aged populations. HIV patients with AIDS are younger and therefore, comparisons have to be carried out with appropriately age-matched controls.

The cause of this hypogonadism is probably as a result of a number of factors, including lipodystrophy induced by highly active retroviral medications; testicular atrophy caused by opportunistic infection; disruption of the HPG axis resulting from malnutrition; and the results of medications such as the antimycotic ketoconazole, which inhibits steroid biosynthesis (17).

## Clinical presentation and diagnosis

### Symptoms

There are a number of symptoms and signs related to low testosterone concentrations that indicate a diagnosis of hypogonadism. The clinical practice guidelines by the Endocrine Society (2) and the AACE (3) suggest that physicians should measure the testosterone levels of men with any of the symptoms and signs outlined in Table 5.

**Table 5:** Symptoms and signs of hypogonadism (2,99)

**Primary symptoms**
Reduced libido
Lack of effect of PDE5 inhibitors for erectile dysfunction
Reduced muscle mass and strength
Depressed mood
Decreased energy or vitality; increased fatigue
Osteoporosis or low bone mass
**Other symptoms and signs suggestive of low testosterone**
Incomplete sexual development, eunuchoidism, aspermia
Decreased spontaneous erections
Breast discomfort, gynaecomastia
Loss of body (axillary and pubic) hair; reduced shaving
Very small or shrinking testes (especially < 5 ml)
Inability to father children; low or zero sperm counts(oligospermia or azoospermia)
Low trauma fracture, low bone mineral density
Poor concentration and memory
Sleep disturbance; increased sleepiness
Mild anaemia (normochromic, normocytic, in the female range)
Increased body fat, body mass index
Diminished physical or work performance
Menopausal-type hot flushes

Screening tools can be helpful in identifying patients with a high probability of having low testosterone. The Androgen Deficiency in Ageing Males (ADAM) questionnaire can be helpful to initiate a conversation about the symptoms they may be experiencing (75) (Table 6). It is important to note that screening questionnaires such as ADAM should never be used in isolation to diagnose clinical hypogonadism.

**Table 6:** Androgen deficiency in ageing males (ADAM) questionnaire (75)

1.Do you have a decrease in libido (sex drive)?
2.Do you have a lack of energy?
3.Do you have a decrease in strength and/or endurance?
4.Have you lost height?
5.Have you noticed a decreased enjoyment of life?
6.Are you sad and/or grumpy?
7.Are your erections less strong?
8.Have you noticed a recent deterioration in your ability to play sports?
9.Are you falling asleep after dinner?
10.Has there been a recent deterioration in your work performance?
If the answer is ‘yes’ to question 1 or 7, or at least 3 of the other questions, low testosterone may be present.

The symptoms most associated with hypogonadism are low libido, erectile dysfunction (ED), decreased muscle mass and increased body fat, decreased BMD, decreased vitality, and depressed mood. None of these symptoms is unique to hypogonadism, so one or more of these symptoms must be combined with a low testosterone concentration for the diagnosis to be made.

Although the Endocrine Society does not suggest routine screening of non-symptomatic at-risk patients, physicians need to be aware of the high prevalence of low testosterone with other conditions, discussed earlier in this review, such as type 2 diabetes, HIV, and COPD, as well as end-stage renal disease, which are all high risk factors for hypogonadism (Table 7). In our opinion, screening for at-risk patients is therefore worthy of consideration.

**Table 7:** Conditions requiring measurement of serum testosterone (as suggested by the Endocrine Society) (2)

Infertility
Osteoporosis, low trauma fracture
Type 2 diabetes mellitus
Glucorticoid, ketoconazole, opioid or other medications that affect T metabolism or production
Moderate to severe COPD
Sellar mass, radiation to the sellar region, or other diseases of the sellar region
End-stage renal disease, maintenance haemodialysis
HIV-associated weight-loss

COPD, chronic obstructive pulmonary disease.

### Diagnosis

**Figure 3:**
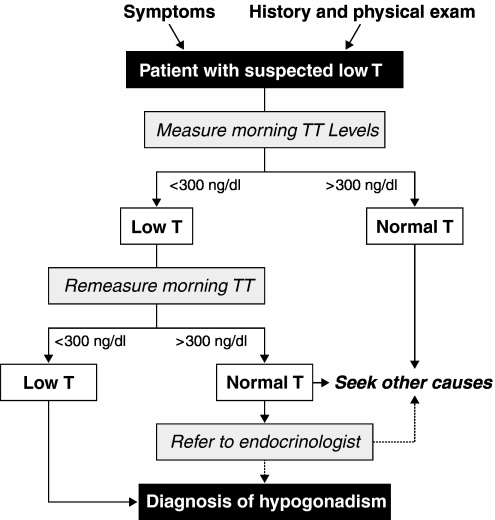
An algorithm for the diagnosis of hypogonadism (2,76,77). TT, total testosterone

Figure 3 shows an algorithm for the diagnosis of hypogonadism (2,76,77). The Endocrine Society recommends that the diagnosis of testosterone be made in men who have both consistent signs and symptoms and low total testosterone levels. Serum total testosterone is the easiest and most straightforward measurement to take for the first measurement. Most hospital laboratories can provide total testosterone measurements of good accuracy and reliability. As testosterone is subject to circadian and circannual rhythms it is recommended to draw the blood sample in the morning. There are no absolute testosterone levels below which a man can unambiguously be stated to be hypogonadal. The Endocrine Society recommends 300 ng/dl (10.4 nmol/l) as a good level to consider as the lower limit of normal total testosterone. The AACE suggests 200 ng/dl (3), and the International Society of Andrology (ISA), International Society for the Study of Ageing Male (ISSAM), European Association of Urology (EAU), European Academy of Andrology (EAA), American Society of Andrology (ASA) recommendations suggest 230 ng/dl is a limit below which patients will usually benefit from testosterone replacement treatment (4).

Total testosterone represents the total of free, SHBG-bound, and albumin-bound testosterone. SHBG levels are easily affected by many conditions, so total testosterone measurements can therefore be misleading indicators of hypogonadism. Table 8 gives a list of conditions when SHBG levels may be higher or lower than normal. As obese or elderly men are not uncommon in routine clinical practice, it is prudent not to rely on total testosterone concentrations for diagnosing low testosterone concentrations for these patients. We therefore recommend that you should consider free testosterone or BAT measurements in all men other than healthy lean young men (whose SHBG levels are presumably normal and whose measured total testosterone concentration is reliable).

**Table 8:** Conditions with high and low SHBG levels (2,76)

Increased SHBG concentrations	Decreased SHBG concentrations
Ageing	Hypothyroidism
Hepatic cirrhosis	Obesity; metabolic syndrome
Use of anticonvulsants	Type 2 diabetes
Use of estrogens	Nephrotic syndrome
Hyperthyroidism	Use of glucocorticoids, progestins, anabolic steroids
HIV infection	
Catabolic conditions (malnutrition; malabsorption)	

SHBG, sex hormone binding globulin.

It is important that physicians use reliable laboratories and that they are aware of their reference ranges for testosterone. For a primary care physician, it might be considered appropriate to refer a patient who requires further testosterone tests to an endocrinologist. If referral is not possible, one can calculate free testosterone reliably by using total testosterone, albumin and SHBG concentrations using an online calculator (http://www.issam.ch/freetesto.htm) or measure free testosterone accurately in a laboratory by equilibrium dialysis. As with total testosterone measurements, there is no general agreement as to what constitutes the lower limit of normal free testosterone levels, but the Endocrine Society mentions 50 pg/ml for free testosterone measured by equilibrium dialysis and the ISA, ISSAM, EAU, EAA and ASA recommend 65 pg/ml for calculated free testosterone.

The ISA/ISSAM/EAU/EAA/ASA guidelines suggest that subjects with total testosterone levels falling between 230 and 350 ng/dl (8–12 nmol/l) could benefit from having a repeat measurement of total testosterone together with a measurement of SHBG concentrations so as to calculate free testosterone levels, or free testosterone levels can be measured directly via equilibrium analysis in these cases (4).

The final step in determining whether a patient has primary or secondary hypogonadism is measuring the serum LH and FSH. Elevated LH and FSH levels suggest primary hypogonadism, whereas low or low-normal LH and FSH levels suggest secondary hypogonadism. Normal LH or FSH levels with low testosterone suggest primary defects in the hypothalamus and/or the pituitary (secondary hypogonadism). Unless fertility is an issue, it is usually not necessary to measure FSH and determining LH levels alone is sufficient. In secondary hypogonadism, prolactin levels should be obtained to rule out prolactinoma and screening for hemochromatosis should be considered. Pituitary imaging studies to evaluate for structural lesions in the hypothalamic–pituitary region are recommended if the total testosterone concentration is < 150 ng/dl (2). A karyotype should be considered in a young teenager or infertile man with primary hypogonadism to diagnose Klinefelter syndrome (2–4).

## Treatment

### Testosterone formulations

There are a number of options for testosterone replacement therapy in hypogonadal patients and these are summarised in Table 9 (2,78,79).

**Table 9:** Testosterone formulations available in the United States

Formulation	Dose
Intramuscular injections (testosterone enanthate or testosterone cypionate)	75–100 mg weekly or 150–200 mg every 2 weeks
Transdermal patches (non-scrotal)	2.5–7.5 mg applied nightly for 24 h
Transdermal gels	5–10 g applied daily to upper arms/shoulders, or abdomen (5–10 mg testosterone systemically absorbed)
Buccal tablets	30 mg tablet applied to the buccal mucosa every 12 h
Subcutaneous pellets	6–10, 75 mg pellets implanted subcutaneously every 4–6 months
Oral capsule or tablet (methyl testosterone)	Should *not* be used for the treatment of hypogonadism because of risk of liver toxicity and hepatocellular carcinoma

When looking at the treatment options, it is important to keep in mind that the goal of testosterone replacement therapy is to increase blood testosterone concentrations to the normal (eugonadal) range and to match the most appropriate treatment to the individual patient. The eugonadal range for adult men has been considered in most studies to be in the range of 300–1000 ng/dl (the AACE recommends 280–800 ng/dl), and it is usually considered best to aim for a testosterone level in the mid-normal range, avoiding excessive supraphysiological peaks (3,67,79).

#### Intramuscular injections

Testosterone injections have been available for at least 50 years and are usually the cheapest choice for treatment. The testosterone esters, testosterone enanthate or testosterone cypionate, are administered in the office or at home by the patient or a designate. A characteristic of injected testosterone esters is that, after the injection, the serum testosterone levels rise to supraphysiological levels, after which they gradually decline into the hypogonadal range by the end of the dosing interval. Some patients show parallel variations in breast tenderness, sexual activity, emotional stability (anger or depression) and general well-being (fatigue) as the testosterone levels change over time. The usual frequency of injections is once every 2 weeks. The oscillations in the serum testosterone levels can be reduced by increasing the frequency of injections to once a week. However, in some cases, to obtain testosterone concentrations continuously in the normal range would require unacceptably frequent injections of small doses. Mood and sexual function fluctuations can be reduced by starting with lower doses and titrating upward.

A long-lasting formulation of testosterone undecanoate, another testosterone ester, is available in the EU and other countries, but not yet in the US. This is a depot preparation that requires only four injections a year (80) and has a superior pharmacokinetic profile compared with the other injectable testosterone formulations.

#### Transdermal patches

These are applied in the night and provide a good approximation of normal circadian plasma testosterone levels. The scrotum has approximately a 40-fold higher rate of absorption than the forearm and the first testosterone patches were placed on the scrotum, but these are not so popular because the scrotum has to be shaved and the adherence is not so good. Non-genital patches are applied once a day in the evening to the back, abdomen, thighs or upper arms. Skin irritation can occur and a preapplication of a corticosteroid cream to the skin can help reduce it. Rarely, skin lesions are also known to occur following the use of these patches.

#### Transdermal gels

These are colourless hydroalcoholic gels of 1% testosterone. They are applied once a day to the upper arms and shoulders or to the abdomen. Gels with a higher concentration of testosterone are not yet available in the US, but a 2% testosterone gel is available in the EU (81,82). These allow the patient to reduce the total volume of gel applied by about half compared with a 1% gel.

The gel has a number of advantages including ease of use, lower incidence of skin irritation compared with the patch, invisibility of application and flexibility of dosing. An issue with gels is that testosterone can be transferred from the patient to his partner or to children after skin contact. This risk can be minimised by having patients wash their hands with soap and water after applying the gel, by covering the site of application with clothing after the gel has dried, and by washing the application site when skin-to-skin contact is expected.

#### Buccal tablets

These are adhesive tablets containing testosterone that are applied to the gum just above the incisor teeth. They release testosterone slowly, allowing for absorption through the gum and cheek surfaces. In this manner they bypass first-pass hepatic metabolism. The tablet must remain in the mouth for a full 12 h and two are needed for the 24 h dosing period. The incidence of adverse effects is low, although gum and buccal irritation, and alterations in taste have been reported.

#### Subcutaneous pellets

These are amongst the earliest effective formulations for administering testosterone, dating back to the 1940s. Although not frequently used, they remain available. The testosterone pellets are usually implanted under the skin of the lower abdomen using a trochar and cannula or are inserted into the gluteus muscle. Six to ten pellets are implanted at one time and they last 4–6 months, when a new procedure is required to implant more. Testosterone pellets currently are the only long-acting testosterone treatment approved for use in the United States. As a result of their long-lasting effect and the inconvenience of removing them, it is best to use pellets in men for whom the beneficial effects and tolerance for testosterone replacement therapy have already been established.

#### Oral testosterone tablets or capsules

Orally ingested testosterone is inactivated in the liver. The modified testosterone 17α-methyl testosterone, however, has delayed metabolism in the liver. Although it is an effective oral androgen formulation, it is not recommended as a testosterone therapy for hypogonadism because of its hepatotoxic side effects and its association with long-term development of liver tumours. Oral testosterone undecanoate, however, bypasses first-pass metabolism through its preferential absorption into the lymphatic system. It is generally recognised as safe because of the lack of adverse liver side effects, but it is only available outside the US (83).

### Symptom relief

Studies with hypogonadal men have demonstrated that once testosterone levels are restored to a stable normal range, there is an improvement in libido, sexual function, mood and energy levels relatively early in the course of treatment (78,84–86). Over a period of 6 months there is a reduction in fat body mass and an increase in lean body mass, and an improvement in BMD at the hip and spine. Further improvement in these symptoms may be seen after longer term use (9,29,32,67). Some studies have shown that insulin resistance may also improve following testosterone treatment (87,88). This is generally believed to be as a result of reduction in fat mass after testosterone therapy. Further research on this is warranted and testosterone replacement therapy is not currently indicated for treatment of diabetes or metabolic syndrome.

### Contraindications and precautions

The contraindications and main precautions of testosterone therapy are shown in Table 10.

**Table 10:** Contraindications and precautions for testosterone replacement therapy (2)

**Contraindications**
Male breast cancer
Prostate cancer (known or suspected)
Known or suspected sensitivity to ingredients used in the testosterone delivery systems
**Precautions**
Benign prostatic hyperplasia (BPH); lower urinary tractsymptoms (LUTS)
Oedema in patients with preexisting cardiac, renal, or hepatic disease
Gynaecomastia
Precipitation or worsening of sleep apnoea
Azoospermia; testicular atrophy
Erythrocytosis

The most important contraindications are for prostate and breast cancer. Prostate cancer will be discussed in more detail later, but because androgens may have a role in promoting the growth of cancers in these tissues, the presence or suspected presence of either of these cancers is considered an absolute contraindication for testosterone therapy.

It is recommended to perform a baseline digital rectal examinations (DRE) and a baseline PSA level measurement before starting testosterone therapy for any man, whatever his age (2,89).

Testosterone is also contraindicated in pregnant or breast-feeding women. Therefore, testosterone gel users must consider the possibility of contact with, and therefore testosterone transfer to, a pregnant or breast-feeding woman. Care must, therefore, be taken when prescribing testosterone therapy in the above circumstances. Men using a testosterone gel should be advised by their healthcare provider on the ways of minimising the risk of testosterone transfer to women and children.

It is likely that, because hypogonadism is associated with obesity, many hypogonadal patients will have sleep apnoea. Because testosterone therapy may worsen sleep apnoea in some patients, there is a need to ask patients and their partners about any sleep apnoea symptoms, such as excessive snoring or daytime tiredness, they may have before they start treatment. Treatment should probably be avoided in patients with severe, untreated sleep apnoea (2).

### Monitoring treatment

Once testosterone replacement therapy has started, patients need to be carefully monitored. Table 11 shows the principal monitoring requirements for testosterone therapy as specified by the Endocrine Society (2).

**Table 11:** Endocrine Society Guidelines for the monitoring of testosterone therapy (2)

	Start of treatment (baseline)	Each visit	3 months	Annually	1–2 years
Symptom response		**√**	**√**	**√**	
Adverse events		**√**	**√**	**√**	
Formulation-specific AEs		**√**			
Testosterone levels	**√**		**√**		
Haematocrit*	**√**		**√**	**√**	
BMD of lumbar spine/femoral neck†					**√**
DRE‡	**√**		**√**		
PSA‡	**√**		**√**		

*If haematocrit is > 54%, stop therapy until haematocrit decreases to a safe level, evaluate the patient for hypoxia and sleep apnoea, reinitiate therapy with a reduced dose.

†For patients with osteoporosis or low trauma fracture, consistent with standard of care.

‡After 3 months, perform in accordance with guidelines for prostate cancer screening, depending on the age and race of the patient. Obtain urological consultation under certain conditions.

AEs, adverse events; BMD, bone mineral density; DRE, digital rectal examination; PSA, prostate-specific antigen.

As a result of the concerns about prostate cancer it is important to monitor PSA levels and perform a DRE regularly during the course of treatment.

At 3 months and 1 year after starting therapy, the clinical response of testosterone should be evaluated by documenting serum testosterone levels, monitoring serum PSA levels, and performing a DRE. From then on PSA levels need to be checked according to the usual guidelines for prostate cancer screening. Current American Cancer Society guidelines suggest prostate cancer screening on an annual basis for any male over 50 years, for African American males over 45 years, and for higher risk males over 40 years (multiple first-degree relatives affected at an early age) (90). Any significant increase in PSA deserves a referral to a urologist and treatment should be discontinued until evaluated.

Testosterone is a known stimulant of erythropoiesis, so a rise in haematocrit is not unusual and it needs to be monitored. Erythrocytosis incidence is between 3% and 18% with transdermal formulations and up to 44% with current intramuscular injections. Elevated haematocrit values above 54% require action – usually therapy should be stopped until the values decrease to a safe level.

Bone mineral density measurement should also be carried out at baseline because hypogonadism is an important cause of male osteoporosis. BMD measurement in a male with osteopenia/osteoporosis or low trauma fracture can be repeated in 1–2 years after testosterone replacement therapy is initiated (2,79).

#### Benign prostate hyperplasia

Patients with benign prostatic hyperplasia (BPH) treated with androgens are at an increased risk for worsening of signs and symptoms of BPH. The development of BPH requires androgens, but many studies have failed to show an association with testosterone treatment. Prostate volume does, however, increase during testosterone therapy usually in the first 6 months, but this is usually to the normal volume seen in eugonadal men. The correlation of voiding symptoms and prostate size is poor, so there may not be any changes in urine flow rates and prostate voiding symptoms. However, the increase in size of the prostate needs to carefully monitored, and the patient needs to be made aware that there might be increased voiding symptoms during treatment (2,4,9,79,89).

#### Prostate cancer

One of the striking things about a study published in 2007 was that physicians’ number one fear about initiating testosterone therapy was their perception that it increases the risk of prostate cancer. More than 68% of physicians from a worldwide sample associated testosterone replacement therapy with more risks than benefits. The authors demonstrated that, as a result of this, approximately 35% of hypogonadal patients did not receive treatment (20).

The origins of this concern date back to papers published in 1941 that reported that androgens stimulated prostate cancer, whereas oestrogen or castration reduced them (91). Case reports of occult cancers apparently stimulated to become clinically relevant cancers by testosterone treatment added to the concern. In addition, 5 alpha-reductase inhibitors, such as finasteride and dutasteride, reduce prostate volume and PSA levels. This is consistent with a role for testosterone in prostate growth.

However, recent analysis has shown that, although there are case studies of occult conversions, these represent a very small number of the 200,000 cases of prostate cancer diagnosed in the United States and there is no evidence of causality. In fact, prostate cancer is correlated with age in men, and older men tend to have less, not more, testosterone. Likewise, studies have shown that there is no significant difference between testosterone levels in men with or without prostate cancer (89,92). So, at the present time, there is a lack of conclusive evidence that testosterone therapy in hypogonadal men increases the risk of prostate cancer, and there is no evidence that it will promote subclinical cancer to metastatic cancer. There is evidence, however, that testosterone will stimulate the growth of existing prostatic cancers and, of course, existing prostate cancer is contraindicated for testosterone therapy (4). A fuller explanation may be that prostate cancer is very sensitive to changes in serum testosterone when at low concentrations, but is insensitive at higher concentrations because of saturation of the androgen receptors (93).

It is important to mention that the occurrence of prostate cancer in patients with type 2 diabetes is lower than that seen in the general population. There is also the evidence that PSA concentrations are lower in type 2 diabetes patients and related to testosterone concentrations (46,94). It is not yet known if the normal PSA reference ranges should be lowered for men with type 2 diabetes.

The Institute of Medicine has suggested that to assess a moderate increase in the risk of prostate cancer from testosterone treatment, a large trial requiring 5000 men followed over 3–5 years would be needed (95). To date a trial like this has not been performed.

The American Urological Association (AUA) suggests obtaining urological consultation for any of the following: if the patient’s serum PSA concentration exceeds 4 ng/ml; there is a significant rise from one test to the next; or there is an abnormal DRE result (96). There is, however, no established consensus about what constitutes a significant rise in PSA levels or when urological referral should occur for men with normal PSA levels at baseline. An age-related guideline for referral if PSA levels exceed 2.5 ng/ml in males under 60 years or 4 ng/ml in men over 60 years is often used (97). Usually a velocity change of 0.75 ng/ml or greater in a year is a reason to refer (96).

## Conclusion

Hypogonadism is a common condition in the male population. Its high prevalence in older men, the obese and in men with metabolic syndrome, and type 2 diabetes makes it likely that primary care physicians meet these patients in their clinics every day. It is therefore important that physicians are aware of the major symptoms of the condition and of the treatment options currently available. It is important to consider treating symptomatic patients and not leave them untreated because of anxiety over possible adverse events from testosterone replacement therapy, after discussing with them the potential benefits and risks of treatment.
